# USP39-Mediated Non-Proteolytic Control of ETS2 Suppresses Nuclear Localization and Activity

**DOI:** 10.3390/biom13101475

**Published:** 2023-10-01

**Authors:** Yunsik Choi, Yuri Lee, Jin Seo Kim, Peijing Zhang, Jongchan Kim

**Affiliations:** 1 Department of Life Sciences, Sogang University, Seoul 04107, Republic of Korea; 2 Department of Biological Pharmaceutics, Huazhong University of Science and Technology, Wuhan 430074, China

**Keywords:** ETS2, USP39, deubiquitinase, transcription factor

## Abstract

ETS2 is a member of the ETS family of transcription factors and has been implicated in the regulation of cell proliferation, differentiation, apoptosis, and tumorigenesis. The aberrant activation of ETS2 is associated with various human cancers, highlighting its importance as a therapeutic target. Understanding the regulatory mechanisms and interacting partners of ETS2 is crucial for elucidating its precise role in cellular processes and developing novel strategies to modulate its activity. In this study, we conducted binding assays using a human deubiquitinase (DUB) library and identified USP39 as a novel ETS2-binding DUB. USP39 interacts with ETS2 through their respective amino-terminal regions, and the zinc finger and PNT domains are not required for this binding. USP39 deubiquitinates ETS2 without affecting its protein stability. Interestingly, however, USP39 significantly suppresses the transcriptional activity of ETS2. Furthermore, we demonstrated that USP39 leads to a reduction in the nuclear localization of ETS2. Our findings provide valuable insights into the intricate regulatory mechanisms governing ETS2 function. Understanding the interplay between USP39 and ETS2 may have implications for therapeutic interventions targeting ETS2-related diseases, including cancer, where the dysregulation of ETS2 is frequently observed.

## 1. Introduction

The ETS family is a large, evolutionarily conserved group of transcription factors characterized by a common ETS DNA binding domain. In humans, twenty-eight ETS family transcription factors have been identified to date. These factors are involved in regulating various biological processes, including cell cycle control, differentiation, and angiogenesis [1]. The dysregulation of ETS factors has been frequently documented in human tumorigenesis, contributing to tumor initiation, progression, and metastasis [2].

ETS2, a pivotal member of the ETS family of transcription factors, plays a crucial role in various cellular processes, including cell proliferation, differentiation, apoptosis, and tumorigenesis [3,4,5,6,7,8,9,10]. Understanding the regulatory mechanisms and identifying the interacting partners of ETS2 is of utmost importance due to its intricate involvement in these fundamental cellular events. The aberrant activation of ETS2 is linked to a range of human cancers, underscoring its significance as a promising therapeutic target for ETS2-related diseases [2]. Therefore, investigating the interplay between ETS2 and its regulatory components becomes crucial for elucidating the precise role of ETS2 in cellular processes and developing innovative strategies to modulate its activity.

ETS family members are known to undergo extensive post-translational modifications such as phosphorylation, acetylation, SUMOylation, and ubiquitination [11]. These post-translational modifications can regulate the activity of ETS factors by affecting DNA binding, nucleocytoplasmic shuttling, and protein stability [11]. ETS2, for example, has been reported to undergo phosphorylation, leading to increased transcriptional activity and protein stability [12,13]. Additionally, prior studies have revealed that both ETS2 and its paralog, ETS1, undergo ubiquitination mediated by COP1, resulting in the facilitated degradation of ETS1 and ETS2 [14,15].

Deubiquitinases (DUBs) are key players in the dynamic control of protein ubiquitination, and their ability to modulate protein stability, localization, and activity has profound effects on cellular processes [16]. ETS1 was identified as a target of USP9X with NRAS regulatory potential in a USP9X ubiquitylome analysis [17]. Furthermore, USP7 has more recently been recognized as a DUB for ETS2, which was predicted as a substrate protein through the analysis of publicly available proteomic databases [18]. In both studies, the DUBs regulated protein stability by inhibiting the proteasomal degradation of ETS1 and ETS2. The central objective of this study, however, was to uncover fresh perspectives on the regulatory network controlling ETS2. This was achieved by identifying a DUB and delving into its functional role in relation to ETS2 using a human DUB library.

In this study, we attempted to identify a DUB that interacts with and deubiquitinates ETS2. Our aim was to enhance our understanding of ETS2-mediated transcription and make a contribution to the development of innovative strategies for modulating ETS2 activity. This was achieved by investigating the regulatory mechanisms and functional implications of the interaction between the DUB and ETS2.

## 2. Materials and Methods

### 2.1. Cell Culture

HEK293T (Human embryonic kidney 293) cells were obtained from the American Type Culture Collection (ATCC; Manassas, VA, USA). The cells were maintained in Dulbecco’s Modified Eagle’s Medium (DMEM) (GenDepot; Katy, TX, USA) supplemented with 10% fetal bovine serum (FBS) and 1% penicillin–streptomycin.

### 2.2. Plasmids

The open reading frame sequence of ETS2 was cloned into a pDONR vector (Gateway entry vector) and subsequently subcloned into an MYC peptide-tagged expression vector using the Gateway cloning system (Thermo Fisher Scientific; Waltham, MA, USA). The open reading frame sequences of human DUBs were cloned as previously described [19]. To create a pGL3-LAIR1-p6 vector, an ETS2-responsive luciferase reporter construct, the p6 promoter region of the LAIR1 (Leukocyte Associated Immunoglobulin Like Receptor 1) gene was amplified from HaCaT cell genomic DNA (kindly provided by Dr. Bong Gun Ju at Sogang University) and inserted into a pGL3 luciferase vector, following the methods described in the previous study [20]. An HA-tagged ubiquitin plasmid was obtained from Addgene (Watertown, MA, USA), and a pRL (Renilla luciferase) vector was purchased from Promega (Madison, WI, USA).

### 2.3. Immunoblotting

Immunoblotting was performed as described [19]. Cell lysis was carried out using RIPA buffer supplemented with protease and phosphatase inhibitors (GenDepot; Houston, TX, USA). Subsequent to cell lysis, SDS-PAGE was employed to separate the proteins, which were later transferred onto a PVDF membrane. Blocking was performed using 5% non-fat milk within Tris-buffered saline-Tween 20 (TBS-T), followed by an incubation with specific primary antibodies. After undergoing TBS-T washing, the membranes were treated with an HRP-conjugated secondary antibody. Detection of the bands was achieved through chemiluminescence. The following primary antibodies were used: anti-MYC (1:10,000; #60003-2-Ig; Proteintech; San Diego, CA, USA), anti-FLAG (1:2000; #20543-1-AP; Proteintech; San Diego, CA, USA), anti-HSP90 (1:1000; #sc-69703; Santa Cruz Biotechnology; Santa Cruz, CA, USA), anti-Cyclophilin B (1:20,000; #PA1-027A; Thermofisher Scientific; Waltham, MA, USA), anti-HA (1:1000; #sc-7392; Santa Cruz Biotechnology; Santa Cruz, CA, USA), anti-USP39 (1:4000; #23865-1-AP; Proteintech; San Diego, CA, USA), anti-Lamin B1 (1:4000; #12987-1-AP; Proteintech; San Diego, CA, USA), and anti-α-tubulin (1:10,000; #66031-1-Ig; Proteintech; San Diego, CA, USA).

### 2.4. Site-Directed Mutagenesis

To generate truncation mutants of ETS2 and USP39, we utilized the EZchange site-directed mutagenesis kit from Enzynomics (Daejeon, Korea), following the manufacturer’s protocol. Specifically, four truncation mutants of ETS2 lacking amino acid residues 171–469 (PNT-only), 363–469 (ΔETS), 85–170 (ΔPNT), and 1–170 & 363–469 (171–362) and two truncation mutants of USP39 lacking amino acid residues 220–565 (ΔUSP) and 103–200 (ΔZNF) were created.

### 2.5. Immunoprecipitation and Pulldown Assays

At 48 h post-transfection, the cells were lysed using NETN buffer (200 mM Tris-HCl, pH 8.0, 100 mM NaCl, 0.05% Nonidet P-40, 1 mM EDTA) supplemented with protease and phosphatase inhibitors (GenDepot, Houston, TX, USA). For the isolation of SFB-tagged proteins, cell lysates were treated with S-protein beads (Merck, Darmstadt, Germany). Similarly, for the capture of MYC-tagged proteins, the cell lysates were exposed to MYC-beads (Thermofisher, Waltham, MA, USA). Following an overnight inversion at 4 °C, the precipitated protein complexes underwent triple washes with NETN buffer. The bound proteins were then eluted by boiling them with 2× Laemmli buffer and subjected to vortexing for 30 s. Subsequently, the eluates were subjected to immunoblotting using the specified antibodies.

### 2.6. RNA Interference

siRNA oligonucleotides were purchased from Genolution (Seoul, Korea). For siRNA transfection, X-tremeGENE siRNA Transfection Reagent (Merck, Darmstadt, Germany) was used following the manufacturer’s protocol. The target sequences of oligos B and C for USP39 mRNA are as follows:

oligo B: CAAUGAUUAUGCCAACGCU.

oligo C: CUCGAAAUUUCAAGGCACA.

### 2.7. Deubiquitination Assays

HEK293T cells were co-transfected with MYC-ETS2, SFB-USP39/SFB-GFP, and HA-ubiquitin constructs and treated with the proteasome inhibitor MG132 (10 μM) for 6 h. When examining endogenous ubiquitination, the HA-ubiquitin vector was omitted. Cells were harvested and lysed in RIPA buffer (50 mM Tris-HCl at pH 7.4, 150 mM NaCl, 1% Nonidet P-40, 0.5% sodium deoxycholate, 1 mM EDTA) containing 1% SDS for denaturation. Cell lysates were heated at 95 °C for 5 min in the presence of 1% SDS, followed by a 10-fold dilution with RIPA buffer (to 0.1% SDS) and sonication. ETS2 was immunoprecipitated with anti-MYC beads and then subjected to immunoblotting using the specified antibodies.

### 2.8. Cycloheximide Chase Assay

HEK293T cells were seeded in 10 cm dishes and co-transfected with MYC-ETS2, MYC-GFP (as an equal transfection control) and SFB-USP39 or SFB-GFP. After 24 h, cells were transferred to a 6-well plate. Then, cells were treated with cycloheximide (CHX) at a final concentration of 100 μg/mL to inhibit protein synthesis at the indicated time points. The cell lysates were prepared by resuspending cells in RIPA buffer containing protease inhibitors and phosphatase inhibitors. Equal amounts of protein were loaded onto SDS-PAGE gels for immunoblotting analysis. MYC-ETS2 protein levels were quantified using the image processing software ImageJ (version 1.53t) (accessed on 1 May 2023. https://imagej.nih.gov/ij/index.html) by normalizing them to MYC-GFP levels.

### 2.9. Luciferase Assays

HEK293T cells were seeded in 24-well plates and co-transfected with the pRL (Renilla luciferase), the pGL3-LAIR1-p6 and SFB-USP7/SFB-USP39/SFB-GFP vectors with or without MYC-ETS2. To knock down endogenous USP39, siRNA oligonucleotides were included for transfection instead of the SFB-GFP, SFB-USP39, and MYC-ETS2 vectors. After 48 h, cells were lysed, and luciferase activity was measured using the Dual-Luciferase Reporter Assay System (Promega; Madison, WI, USA) according to the manufacturer’s protocol. Renilla luciferase activity was used as an internal control to normalize the firefly luciferase activity.

### 2.10. Cytoplasmic and Nuclear Fractionation Assays

HEK293T cells were seeded in 6 cm dishes and co-transfected with SFB-USP39/SFB-GFP and MYC-ETS2. After 48 h, cells were harvested, and cytoplasmic and nuclear fractions were prepared using the Nuclear Extraction Kit (EMD Millipore Corporation; Burlington, MA, USA) according to the manufacturer’s protocol. Equal amounts of protein were loaded onto SDS-PAGE gels for immunoblotting analysis.

### 2.11. Gene Expression Omnibus (GEO) Repository Dataset Analysis

We downloaded an RNA sequencing dataset from the NCBI Gene Expression Omnibus (GEO) repository, specifically GSE157365. Within the GSE3678 dataset, we conducted a gene expression comparison involving three replicates each of control and USP39 knockdown A2780 cells, a human ovarian cancer cell line. Initially, we identified 4777 differentially expressed genes (*p* < 0.05) in response to USP39 knockdown. Subsequently, we compiled a list of 206 ETS2 target genes from the CHEA Transcription Factor Targets dataset, sourced from the Ma’ayan Lab homepage (accessed on 2 September 2023. https://maayanlab.cloud/Harmonizome/). Of these 206 genes, 72 displayed significant alterations in gene expression within USP39-KD cells. Specifically, 42 genes exhibited up-regulation, while 30 genes were down-regulated in response to USP39 knockdown. For our analysis, we converted the gene expression levels to log10 values and generated scatterplots using the R programming language.

### 2.12. Statistical Analysis

**Figure 1:**
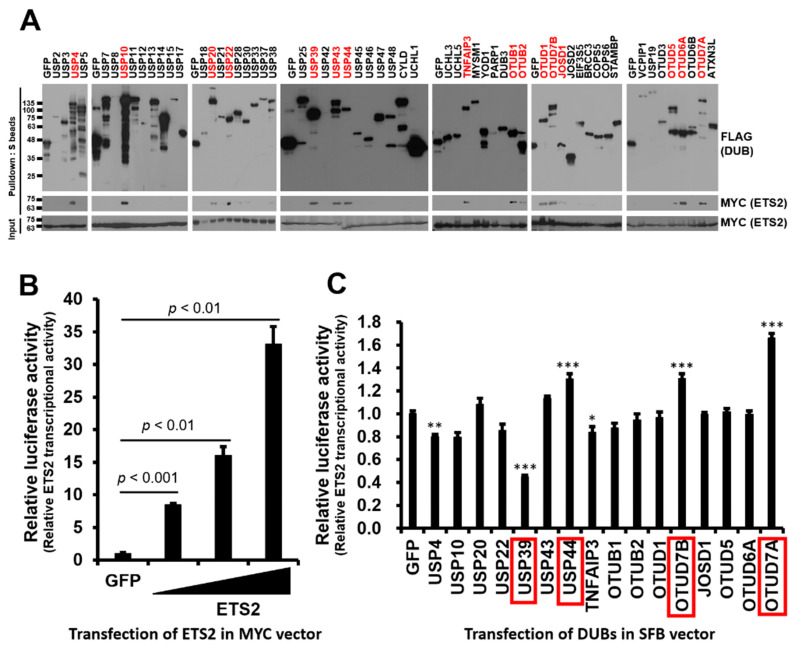
Identification of DUBs interacting with ETS2 and modulating its transcriptional activity. (**A**) HEK293T cells were co-transfected with MYC-ETS2 and SFB-tagged 59 human DUBs and subjected to pulldown using S-protein beads. The pulled-down DUBs and DUB-bound ETS2 were detected via immunoblotting using anti-FLAG and anti-MYC antibodies, respectively. (**B**) An ETS2-responsive luciferase reporter vector, containing the LAIR1-p6 region, was constructed and subsequently validated for its reporter activity. Then, 4 ng, 20 ng and 100 ng of the MYC-ETS2 vector were transfected into each well of a 24-well plate to examine the dose-dependent transcriptional activity of ETS2. An MYC-GFP vector was used as a control. Statistical significance was determined using an unpaired *t*-test. (**C**) Sixteen DUBs interacting with ETS2 were subjected to a reporter assay using the ETS2-responsive luciferase reporter vector. HEK293T cells were co-transfected with the pGL3-pLAIR-p6 vector, pRL (Renilla luciferase) vector, and each SFB-tagged DUB plasmid. Firefly luciferase activity was normalized by Renilla luciferase activity to measure the relative luciferase activity. One-way ANOVA followed by post hoc testing and further screening indicated that four DUBs (highlighted in red boxes) significantly modulated the transcriptional activity of ETS2, with substantial level of significance (*p* < 0.001), resulting in a minimum of 30% up- or down-regulation. The level of significance was set at *p* < 0.05. * *p* < 0.05, ** *p* < 0.005, *** *p* < 0.001. In panels B and C, quadruplicate samples were analyzed for quantitation, and error bars represent the standard error of the mean (s.e.m.).

Data were analyzed using analysis tools, including Microsoft Excel 2016, GraphPad Prism (version 8.0.2) and R (version 4.3.1). Statistical significance, except for the selection of candidate DUBs based on the transcriptional activity of ETS2 in Figure 1C, was determined using an unpaired two-tailed Student’s *t*-test. Differences were considered statistically significant at *p* < 0.05, and the data are presented as mean ± s.e.m. To select candidate DUBs that significantly modulate ETS2′s transcriptional activity, we employed one-way ANOVA followed by post hoc analysis (Tukey’s Honestly Significant Difference (HSD) test). The level of significance was set at *p* < 0.05. After this analysis, we narrowed down four DUBs: USP39, USP44, OTUD7A, and OTUD7B. These DUBs exhibited a substantial level of statistical significance, with a *p*-value lower than 0.001, and demonstrated a modulation of ETS2 transcriptional activity by at least 30% (either up- or down-regulation).

## 3. Results

### 3.1. Identification of DUBs Interacting with ETS2 and Modulating Its Transcriptional Activity

To identify DUBs interacting with ETS2, we conducted binding assays between ETS2 in an MYC-tagged expression vector and DUBs from a human DUB library consisting of 59 DUBs in an SFB-tagged expression vector. We co-transfected HEK293T cells with each SFB-DUB and MYC-ETS2 vectors and pulled down the DUB using S-protein agarose beads. As shown in Figure 1A, sixteen DUBs displayed noticeable interactions with ETS2.

To examine the functional role of the binding DUBs, we constructed an ETS2-responsive luciferase reporter plasmid. Based on Cao et al. [20], the ~250 bp minimal promoter region of the human leukocyte-associated immunoglobulin-like receptor-1 (LAIR1) gene, named p6, demonstrated equivalent responsiveness to the highest responsive promoter region. Therefore, we constructed pGL3-LAIR1-p6 as an ETS2-responsive reporter plasmid and examined transcription activity of ETS2 by transfecting increasing doses of MYC-ETS2 plasmid (4, 20 and 100 ng). As expected, the results showed a dose-dependent responsiveness to MYC-ETS2 (Figure 1B). Using pGL3-LAIR1-p6, we performed a luciferase reporter assay to test whether the transcription activity of ETS2 is regulated by any of the interacting DUBs. We co-transfected pGL3-LAIR1-p6 with each SFB-DUB/SFB-GFP plasmid into HEK293T cells. Among the sixteen DUBs, six (USP4, USP39, USP44, TNFAIP3, OTUD7B and OTUD7A) significantly (one-way ANOVA, *p* < 0.05) modulated the transcriptional activity of ETS2, and four of them (USP39, USP44, OTUD7A, and OTUD7B) exhibited a substantial level of statistical significance with a *p*-value lower than 0.001, while two other candidates had higher *p*-values. Furthermore, four DUBs showed at least a 30% up- or down-regulation of ETS2’s transcription activity (Figure 1C).

### 3.2. USP39 Interacts with and Suppresses Transcriptional Activity of ETS2

**Figure 2:**
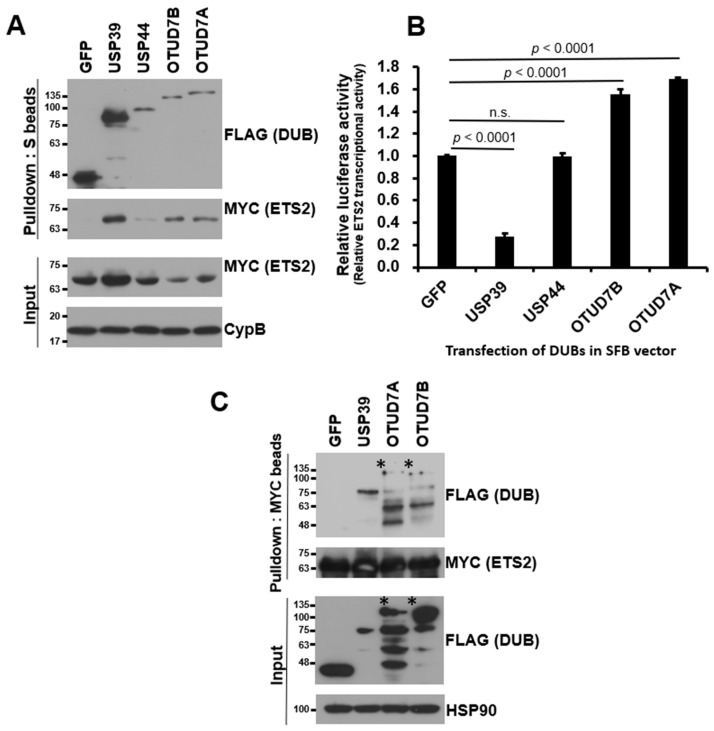
USP39 interacts with and suppresses transcriptional activity of ETS2. (**A**) HEK293T cells were co-transfected with MYC-ETS2 and SFB-tagged four candidate DUBs and subjected to pulldown using S-protein beads. The pulled-down DUBs and DUB-bound ETS2 were detected via immunoblotting using anti-FLAG and anti-MYC antibodies, respectively. Three DUBs, excluding USP44, exhibited a noticeable interaction with ETS2. (**B**) Four DUBs were subjected to a reporter assay using the ETS2-responsive luciferase reporter vector. HEK293T cells were co-transfected with the pGL3-pLAIR-p6 vector, pRL (Renilla luciferase), and each SFB-tagged DUB plasmid. Firefly luciferase activity was normalized by Renilla luciferase activity to measure the relative luciferase activity. Three DUBs, excluding USP44, significantly modulated the transcriptional activity of ETS2. Error bars represent the standard error of the mean (s.e.m.). n.s. indicates “not significant”. Quadruplicate samples were analyzed for quantitation, and statistical significance was determined using an unpaired *t*-test. (**C**) Reverse co-immunoprecipitation demonstrated that USP39 exhibited the strongest interaction with ETS2. The asterisks indicate the weak binding of OTUD7A and OTUD7B to ETS2 compared to the input levels.

To compare the relative binding strength between ETS2 and each candidate DUB, we conducted a small-scale binding assay. We co-transfected HEK293T cells with each SFB-DUB and MYC-ETS2 vectors and pulled down the DUB using S-protein agarose beads. As shown in Figure 2A, USP44 displayed a very weak interaction, while the other three (USP39, OTUD7A, and OTUD7B) showed noticeable interactions with ETS2. Additionally, we compared their influence on the transcriptional activity of ETS2 using a luciferase reporter assay and found that the three DUBs, which exhibited decent interactions with ETS2, consistently and significantly regulated ETS2’s transcriptional activity (Figure 2B). USP39 suppressed the transcription activity of ETS2 by approximately 72.8%, while OTUD7A and OTUD7B increased the transcription activity of ETS2 by approximately 55.2% and 68.5%, respectively. Therefore, we excluded USP44 due to its relatively weak interaction with ETS2 and the minimal change it induced in the transcriptional activity of ETS2.

To validate the binding between ETS2 and the three candidate DUBs, we conducted reverse co-immunoprecipitation using MYC antibody-conjugated agarose beads to pull down MYC-tagged ETS2. As shown in Figure 2C, USP39 exhibited the strongest interaction with ETS2 compared to OTUD7A and OTUD7B. Based on the mutual binding affinity between USP39 and the substrate protein ETS2, as well as its influence on the transcriptional activity of ETS2, we selected USP39 as the candidate DUB for ETS2 and proceeded with further characterization.

### 3.3. ETS2 Interacts with USP39 through the Respective Amino-Terminal Regions

**Figure 3:**
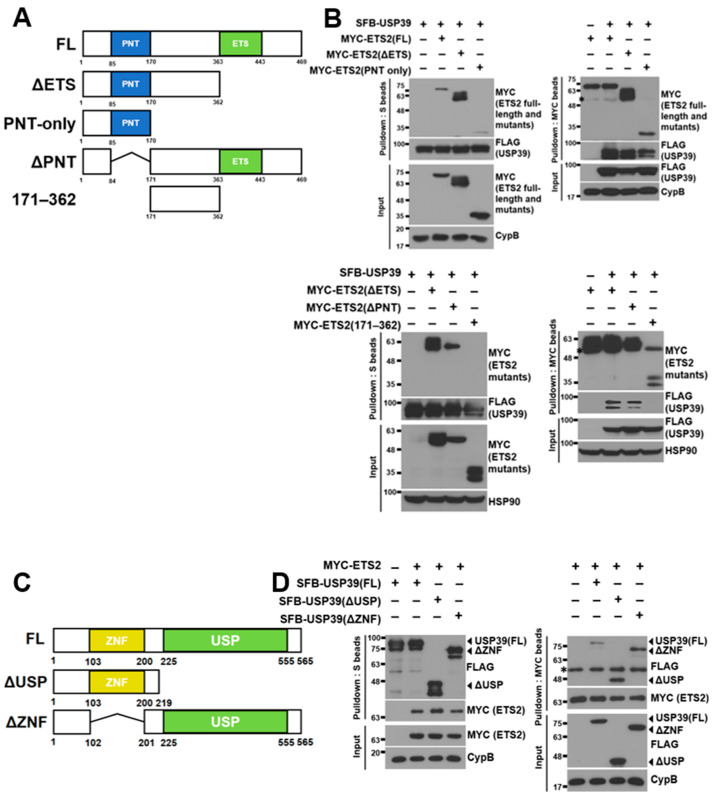
Amino-terminal regions of ETS2 and USP39 are necessary for their interaction. (**A**) Schematic representation of full-length ETS2 and its deletion mutants. ΔETS: ETS domain deleted; PNT-only: Pointed domain containing; ΔPNT: PNT domain deleted; 171–362: amino acids #171–362 containing (**B**) (Top) Full-length USP39 vector was co-transfected with full-length ETS2 or its deletion mutant vectors into HEK293T cells. Either USP39 or ETS2/its mutants were pulled down using S-protein beads or MYC antibody-conjugated agarose beads, respectively. Then, bound ETS2/its mutants or USP39 were detected using anti-MYC or anti-FLAG antibodies, respectively. Cyclophilin B (CypB) was used as the loading control. (Bottom) Full-length USP39 vector was co-transfected with each ETS2 mutant (ΔETS, ΔPNT and 171–362) vector into HEK293T cells. Either USP39 or ETS2 mutants were pulled down using S-protein beads or MYC antibody-conjugated agarose beads, respectively. Then, bound ETS2 mutants or USP39 were detected using anti-MYC or anti-FLAG antibodies, respectively. Heatshock protein 90 (HSP90) was used as the loading control. FL: full-length. The asterisks indicate heavy chains from the anti-MYC antibodies during immunoprecipitation. (**C**) Schematic representation of full-length USP39 and its deletion mutant. ΔUSP: Ubiquitin-specific protease domain deleted; ΔZNF: zinc finger domain deleted. (**D**) Full-length ETS2 vector was co-transfected with full-length USP39 or its deletion mutant vectors into HEK293T cells. USP39/its mutants or ETS2 were pulled down using S-protein beads or MYC antibody-conjugated agarose beads, respectively. Then, bound ETS2 or USP39/its mutants were detected using anti-MYC or anti-FLAG antibodies, respectively. Cyclophilin B (CypB) was used as the loading control. FL: full-length. The asterisk indicates heavy chains from the anti-MYC antibodies during immunoprecipitation.

To identify the domains responsible for the interaction between ETS2 and USP39, we created several deletion mutants of ETS2:ΔETS (ETS domain-deleted, aa 1–362), PNT-only (Pointed domain-containing, aa 1–170), ΔPNT (Pointed domain-deleted, aa 1–84 & 171–469) and 171–362 (containing aa 171–362) (Figure 3A). The ETS domain, characterized as a variant of the winged helix–turn–helix motif, is crucial for DNA binding in ETS family transcription factors [21]. The PNT or Pointed domain is responsible for protein–protein interactions, enabling signal transduction mediated by ETS2 [22]. These constructs, along with full-length SFB-USP39, were co-transfected into HEK293T cells, and SFB-USP39 was pulled down using S-protein agarose beads. Figure 3B shows that both full-length ETS2 and three ETS2 mutants, but not 171–362, interacted with USP39. Additionally, we conducted a reverse immunoprecipitation assay by immunoprecipitating ETS2 and its mutants using MYC antibody-conjugated agarose beads. Then, we examined the interaction of SFB-USP39 with these immunoprecipitated proteins. The results demonstrated that USP39 could bind to ETS2 and all its mutants except 171–362. These findings confirm that USP39 binds to the unspecified amino-terminal region of ETS2, and they indicate that the PNT domain is not required for their interaction.

Subsequently, we generated deletion mutants of USP39, ΔUSP (ubiquitin-specific protease domain-deleted, aa 1–219) and ΔZNF (zinc finger domain-deleted, aa 1–102 & 201–565) (Figure 3C), aiming to determine the ETS2-binding region in USP39. USP domains share a conserved catalytic core, and the majority of these domains facilitate the cleavage of the isopeptide linkage between two ubiquitin molecules [23]. Zinc finger proteins play diverse roles in recognizing and interacting with biological macromolecules such as DNA, RNA, and proteins, as well as associating with membranes [24]. Similarly, we co-transfected full-length and mutant USP39 constructs with full-length MYC-ETS2 into HEK293T cells and pulled down both the full-length and mutant USP39 proteins using S-protein agarose beads. As depicted in Figure 3D, both the full-length USP39 and the mutant forms exhibited interaction with ETS2. In addition, we conducted a reverse immunoprecipitation assay by immunoprecipitating ETS2 using MYC antibody-conjugated agarose beads. We then examined the interaction between ETS2 and USP39 and USP39 mutants. The results demonstrate that ETS2 bound to USP39 and all its mutants. These findings suggest that ETS2 specifically binds to the amino-terminus of USP39 (aa 1–102 and/or 201–219), and the ZNF domain is not required for their interaction.

### 3.4. USP39 Deubiquitinates ETS2 but Does Not Affect Its Protein Stability

**Figure 4:**
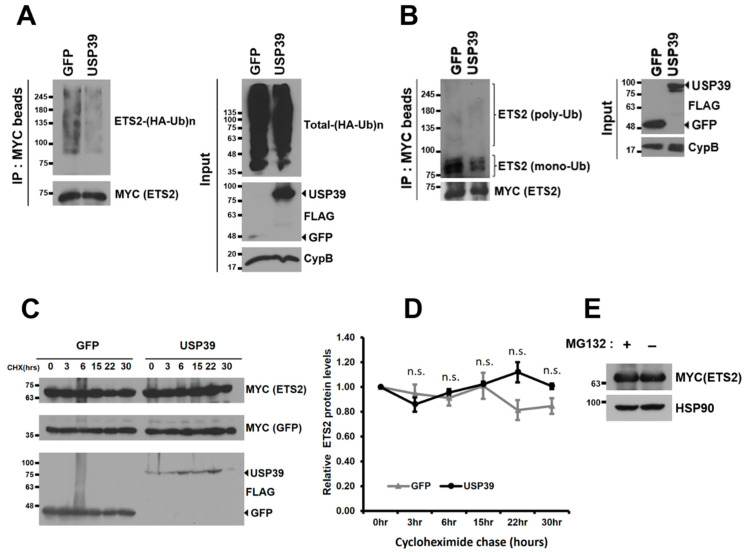
USP39 deubiquitinates ETS2 but does not affect its protein stability. (**A**) HEK293T cells were co-transfected with SFB-GFP/SFB-USP39, MYC-ETS2, and HA-ubiquitin plasmids. After treating the cells with MG132 (10 μM) for 6 h, cell lysates were obtained, and ETS2 was pulled down using MYC antibody-conjugated agarose beads. Immunoblotting was performed using antibodies against HA (to detect ubiquitinated ETS2) and MYC (to detect ETS2). (**B**) HEK293T cells were co-transfected with SFB-GFP/SFB-USP39 and MYC-ETS2 plasmids. After treating the cells with MG132 (10 μM) for 6 h, cell lysates were obtained, and ETS2 was pulled down using MYC antibody-conjugated agarose beads. Immunoblotting was performed using antibodies against Ub (to detect endogenously ubiquitinated ETS2) and MYC (to detect ETS2). (**C**) HEK293T cells were co-transfected with SFB-USP39 or SFB-GFP, MYC-GFP, and MYC-ETS2 plasmids. The cells were treated with the translation inhibitor cycloheximide (100 μg/mL) for the indicated time periods. The protein stability of MYC-ETS2 was examined using anti-MYC antibody. (**D**) The relative protein levels of MYC-ETS2 were quantified by normalizing them to the expression levels of the MYC-GFP control. Error bars represent the standard error of the mean (s.e.m.). n.s. indicates “not significant”. Samples in triplicate at each time point were analyzed for quantitation and statistical significance was determined using an unpaired *t*-test. (**E**) HEK293T cells transfected with MYC-ETS2 were treated with either 10 μM MG132 (+) or a vehicle control (−) for 6 h. Subsequently, the cells were harvested and lysed for immunoblotting. Immunoblotting was performed using antibodies against MYC (to detect ETS2) and heatshock protein 90 (HSP90, used as the loading control).

Since ETS2 is targeted for proteasomal degradation through ubiquitination via the E3 ubiquitin ligase COP1 [8,14,15], we investigated whether USP39 deubiquitinates ETS2 and regulates its protein stability. As depicted in Figure 4A, USP39 noticeably deubiquitinates the polyubiquitin chains of ETS2. We then examined the endogenous ubiquitination of ETS2, which was not previously investigated. Interestingly, ETS2 was primarily mono-ubiquitinated (multiple bands around 75–100 kDa), rather than polyubiquitinated by endogenous ubiquitins (Figure 4B). The pattern of endogenous ubiquitination of ETS2 was comparable to that of other proteins, such as p53 [25] and AT3 [26], both of which demonstrated primarily mono-ubiquitination. USP39 deubiquitinated both the mono- and polyubiquitination of endogenously ubiquitinated ETS2.

Although ETS2 was deubiquitinated by USP39, the protein stability of ETS2 did not show substantial changes over the 30 h period in this study (Figure 4C,D). Furthermore, ETS2 protein was not accumulated when a proteasome inhibitor was treated, suggesting that ETS2 protein is not primarily degraded via the ubiquitin–proteasomal pathway (Figure 4E). The data suggest that the deubiquitinase activity of USP39 negatively regulates ETS2’s transcriptional activity rather than affecting its protein stability.

### 3.5. USP39 Represses the Transcriptional Activity of ETS2 Partly by Suppressing Its Nuclear Localization

**Figure 5:**
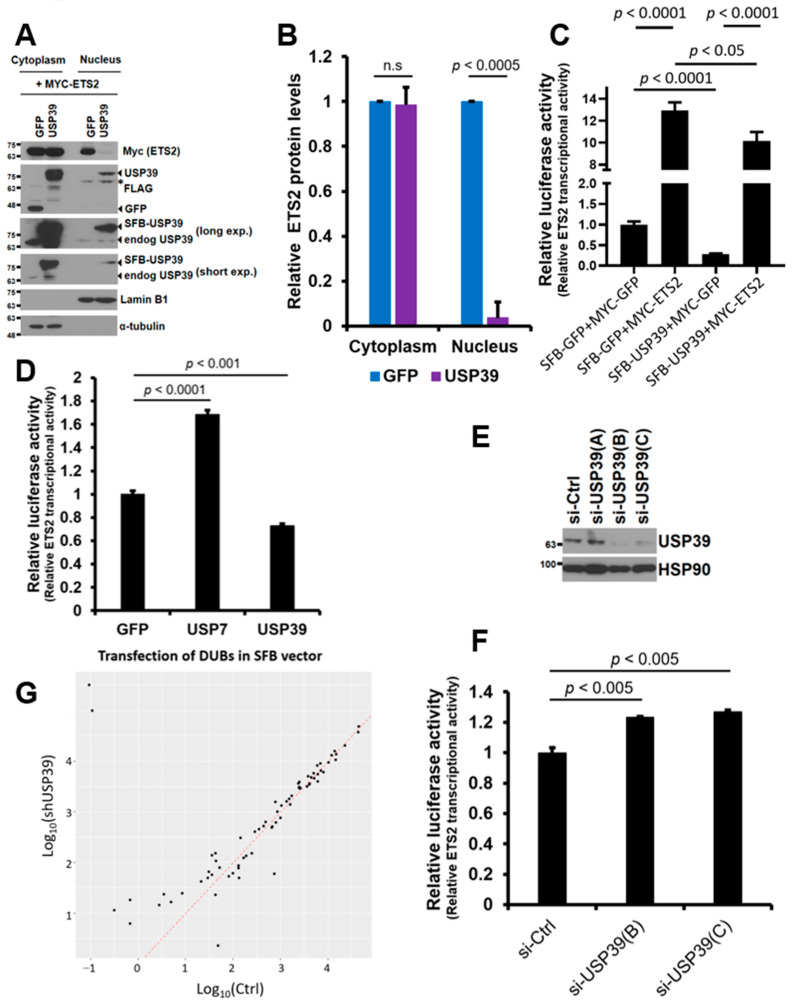
USP39 suppresses the nuclear translocation of ETS2 and opposes the transcription mediated by ETS2. (**A**) HEK293T cells were transiently transfected with MYC-ETS2 and either SFB-GFP or SFB-USP39 plasmids. The cytoplasmic and nuclear fractions of the cell lysates were separated and analyzed via immunoblotting using anti-MYC and anti-FLAG antibodies to detect ETS2 and SFB-USP39/SFB-GFP proteins, respectively. Total USP39, encompassing both endogenous and overexpressed SFB-USP39, was detected using an anti-USP39 antibody. α-tubulin and lamin B1 were used as the loading controls for the cytoplasmic and nuclear fractions, respectively. The asterisk indicates previously blotted bands (lamin B1). (**B**) The cytoplasmic and nuclear ETS2 protein levels were quantified by normalizing them to α-tubulin and lamin B1, respectively. Error bars represent the standard error of the mean (s.e.m.). n.s. indicates “not significant”. Triplicate data were analyzed for quantitation and statistical significance was determined using an unpaired *t*-test. (**C**) MYC-ETS2, SFB-USP39, or both plasmids were co-transfected into HEK293T cells along with pGL3-LAIR1-p6 and pRL (Renilla luciferase) plasmids. Firefly luciferase activity from pGL3-LAIR1-p6 was normalized to Renilla luciferase activity. SFB-GFP and MYC-GFP were used as control vectors. Error bars represent the standard error of the mean (s.e.m.). Quadruplicate samples were analyzed for quantitation and statistical significance was determined using an unpaired *t*-test. (**D**) A similar luciferase assay was performed using SFB-GFP (control vector), SFB-USP7 and SFB-USP39, along with pGL3-LAIR1-p6 and pRL plasmids. Error bars represent the standard error of the mean (s.e.m.). Quadruplicate samples were analyzed for quantitation, and statistical significance was determined using an unpaired *t*-test. (**E**) HEK293T cells were transiently transfected with either control or siRNA oligonucleotides targeting USP39. Cell lysates were then subjected to immunoblotting to assess the efficiency of endogenous USP39 knockdown. Heat shock protein 90 (HSP90) was used as the loading control. (**F**) The indicated siRNA oligonucleotides for USP39 were transfected into HEK293T cells along with pGL3-LAIR1-p6 and pRL (Renilla luciferase) plasmids. Firefly luciferase activity from pGL3-LAIR1-p6 was normalized to Renilla luciferase activity. Error bars represent the standard error of the mean (s.e.m.). Triplicate samples were analyzed for quantitation and statistical significance was determined using an unpaired *t*-test. (**G**) Scatterplots were created for 72 differentially expressed ETS2 target genes (*p* < 0.05) in USP39 knockdown human ovarian cancer cells. These plots were based on log10-transformed expression levels, with control on the x-axis and the USP39 knockdown on the y-axis. The red diagonal line indicates the same levels between the x- and y-axis.

Since USP39 did not affect the protein stability of ETS2, we hypothesized that USP39 may be involved in the subcellular localization of ETS2. To investigate this, we co-transfected MYC-ETS2 with SFB-USP39 or SFB-GFP plasmids into HEK293T cells and separated the cytoplasmic and nuclear fractions of the cell lysates. The protein levels of ETS2 in both fractions were compared via immunoblotting using an anti-MYC antibody. Overexpressing USP39 substantially increased total USP39 levels, including endogenous and overexpressed USP39, in both the cytoplasm and nucleus. Surprisingly, the increased USP39 dramatically and statistically significantly reduced ETS2 protein levels in the nucleus (from 1.00 in GFP control to approximately 0.04 in USP39), while causing only marginal changes in the cytoplasmic fraction (from 1.00 in GFP control to approximately 0.99 in USP39) (Figure 5A,B). This indicates that USP39 strongly suppresses the nuclear localization of ETS2. This finding suggests that USP39-mediated deubiquitination of ETS2 blocks the nuclear translocation of ETS2, resulting in reduced transcriptional activity.

Next, we examined whether USP39 opposes the transcriptional activity driven by ETS2 using a luciferase reporter assay. To do so, we co-transfected the ETS2-responsive reporter plasmid with MYC-ETS2 alone, SFB-USP39 alone, or both plasmids into HEK293T cells. As shown in Figure 5C, ETS2 significantly activated the transcription of the reporter plasmid by approximately 13.0-fold. However, USP39 suppressed the basal levels of transcriptional activity by approximately 71.7%. When the two plasmids were co-expressed, USP39 significantly suppressed the transcription driven by ETS2. This result clearly suggests that USP39 antagonizes the transcriptional activity of ETS2, which may be partly due to the reduced nuclear localization caused by the deubiquitination of ETS2. Recently, Park et al. demonstrated that USP7 deubiquitinates and activates the transcriptional activity of ETS2 [18]. We wondered whether USP7 and USP39 have different effects on ETS2′s transcriptional activity. Interestingly, we observed that USP7 significantly activated ETS2, while USP39 significantly repressed its transcriptional activity (Figure 5D).

In addition, we silenced endogenous USP39 levels to examine its effect on the transcriptional activity of ETS2. Knockdown of USP39 was efficient using siRNA oligos B and C (Figure 5E), and both were used for luciferase reporter assays. When USP39 levels were silenced, the transcriptional activity of ETS2 significantly increased in both knockdown cells (23% and 27% increase in B and C, respectively) (Figure 5F), which implies that USP39 is necessary for suppressing the transcriptional activity of ETS2.

We then examined whether USP39 regulates transcriptional activity using the NCBI Gene Expression Omnibus (GEO) dataset. We analyzed an RNA sequencing dataset from control and USP39 knockdown (KD) human ovarian cancer cells [27]. Among 72 differentially expressed ETS target genes (*p* < 0.05) in USP39-KD cells, we observed a notable prevalence of up-regulated genes (58.3%, 42 genes) compared to down-regulated genes (41.7%, 30 genes) (Figure 5G). This result also partly implicates that USP39 is required for suppressing the transcriptional activity of ETS2.

## 4. Discussion

In the present study, our aim was to identify a DUB that interacts with ETS2, deubiquitinates and functionally regulates it. Comprehensive binding assays utilizing a human DUB library were performed, leading to the identification of USP39 as a novel DUB that strongly interacts with ETS2. Subsequently, we found that USP39 deubiquitinates ETS2 and suppresses its transcriptional activity. The interaction between these two proteins occurs through their unspecified amino-terminal regions of ETS2 and USP39. This suggests that the PNT and zinc finger domains in the amino-terminal regions of ETS2 and USP39 are not necessary for the interaction. However, contrary to our expectations, the deubiquitination mediated by USP39 did not noticeably affect the protein stability of ETS2. Surprisingly, the protein stability of ETS2 remained relatively unchanged, at least within the 30 h period in which we tested it. This suggests that USP39-mediated ubiquitination may impact other aspects of ETS2’s function rather than its stability.

To further explore the mechanism of the USP39-mediated regulation of ETS2, we investigated the nuclear localization of ETS2 in the presence of USP39. Interestingly, we observed that USP39 strongly suppressed the nuclear translocation of ETS2. This finding led us to believe that the inhibition of ETS2’s transcriptional activity by USP39 is at least partly due to its effect on the nuclear localization of ETS2.

In a recent study, Park et al. demonstrated that the suppression of USP7 led to the inhibition of ETS2 deubiquitination and promoted its protein degradation [18]. However, in our initial screening, USP7 was not identified as one of the sixteen ETS2-binding DUBs and, therefore, was excluded from our study, and we did not characterize its function on ETS2. Additionally, the endogenous expression levels of ETS2 were decent and appeared to be relatively unstable within a 12–24 h timeframe in the cell lines tested by the authors [18]. In contrast, in our study, endogenous ETS2 was rarely detectable, possibly due to differences in cellular context, necessitating the examination of overexpressed MYC-ETS2 instead. We found that MYC-ETS2 was highly stable, and its protein degradation was not evident within a 30 h period. Furthermore, we did not observe any notable effect of USP39 on the protein levels of ETS2, prompting us to explore other aspects of deubiquitination-mediated ETS2 regulation.

Interestingly, we discovered that USP39-mediated deubiquitination dramatically suppressed the nuclear localization of ETS2. Although we lack direct evidence on whether the ubiquitination status of ETS2 plays a crucial role in nucleocytoplasmic trafficking, it is well-established that ubiquitination regulates the nuclear import and export of transcription factors [28]. In a recent publication, we reported the catalytic-independent regulation of LEF1 by OTUD7B, wherein OTUD7B promoted the nuclear translocation of LEF1, resulting in increased transcriptional activity [29]. Similarly, USP39 may play a direct role in the cytoplasmic retention of ETS2 through protein–protein interactions. Additionally, It is plausible that USP39 may target and deubiquitinate distinct lysine residues when compared to those targeted by USP7, e.g., M1-, K11-, K27-, and K29-linked polyubiquitination [18], because USP39 and USP7 exhibited the opposite regulation of ETS2 activity. Furthermore, USP39 may deubiquitinate and stabilize unidentified factors that directly induce the cytoplasmic retention of ETS2. Further studies are warranted to investigate the precise mechanism by which the USP39-mediated inhibition of ETS2’s nuclear localization occurs and subsequently leads to the transcriptional suppression of ETS2.

USP39 has been traditionally considered an inactive DUB due to the substitution of the conserved cysteine residue at amino acid 234 in its USP domain with aspartate [30,31,32]. As a result, the deubiquitinase-independent functions of USP39, particularly its role as a splicing factor in pre-mRNA splicing, have been extensively studied [27,33,34,35,36,37]. However, several studies, including our own, have provided evidence that USP39 is fully active and can deubiquitinate and stabilize its substrate proteins, such as β-catenin, SP1, IkBα (a negative regulator of NF-κB), CHK2, STAT1, NAT10, and cyclin B1, regulating various biological functions [38,39,40,41,42,43,44]. These findings suggest that the cysteine residue responsible for enzymatic activity may not be located at position 234 but at another site within the USP domain. In fact, previous studies have shown that the cysteine residue at amino acid 306 is critical for the enzymatic activity of USP39, as its point mutant C306A displayed abrogated deubiquitinase activity [38,39,43]. However, further structural investigation is needed to validate the necessity of C306 or to identify any other critical amino acid residues for the enzymatic activity.

In summary, our study provides valuable insights into the interplay between USP39 and ETS2, expanding our understanding of the regulatory network involving ETS2 and its relevance in disease contexts. Considering the frequent dysregulation of ETS2 observed in various cancers, our findings hold significant potential for therapeutic interventions targeting ETS2-related diseases. Strategies that promote the interaction between USP39 and ETS2 may offer new avenues for precise therapeutic interventions. Moreover, our study highlights the importance of elucidating the molecular mechanisms underlying the regulation of transcription factors, as these insights can contribute to the development of novel therapeutic strategies for diseases driven by dysregulated transcription factors. Further research in this field holds great promise for advancing our understanding of disease pathogenesis and developing effective treatment strategies.

## Data Availability

Not applicable.
